# Banana Classification Using Sanger Sequencing of the Ribosomal DNA Internal Transcribed Spacer (ITS) Region

**DOI:** 10.3390/plants13162173

**Published:** 2024-08-06

**Authors:** Hongyun Zeng, Bingzhi Huang, Linbing Xu, Yuanli Wu

**Affiliations:** Institute of Fruit Tree Research, Guangdong Academy of Agricultural Sciences, Key Laboratory of South Subtropical Fruit Biology and Genetic Resource Utilization, Ministry of Agriculture and Rural Affairs, Guangdong Provincial Key Laboratory of Science and Technology Research on Fruit Tree, Guangzhou 510640, China; zenghongyun@gdaas.cn (H.Z.);

**Keywords:** *Musa*, germplasm resources, genotypes, ITS

## Abstract

Banana (*Musa* spp.) is one of the most economically important horticultural crops. There are many types of banana, with differing ploidy (usually diploid, triploid, or tetraploid) and genome types (most containing the A or/and B genome). Currently, observation and genome type detection are commonly used to identify banana germplasm resources. However, observation is tedious, while genome type detection cannot distinguish categories below genome types. It is, therefore, urgent to establish a simple and effective method for identifying banana germplasm resources. This study sequenced and analyzed the ribosomal DNA internal transcribed spacer (ITS) sequences of 62 banana germplasm resources and found that the sequencing peaks, especially the 20 bp region near the 420-bp position (referred to as the 420-bp region), exhibited relatively recognizable and repeatable polymorphism characteristics. Using the 420-bp region as a marker, we were able to quickly distinguish bananas belonging to different genome type groups or different subgroups in the same genome type group. Moreover, it appeared that Sanger sequencing of ITS could be used to identify hybrid banana offspring. In general, ITS sequencing simplifies the classification of banana germplasm resources and has potential application in several areas of *Musa* improvement.

## 1. Introduction

Banana and plantain (*Musa* spp.) are crops of vital importance to hundreds of millions of people around the world. Most edible banana cultivars are derived from two wild species, namely *Musa acuminata* Colla (A genome) and *Musa balbisiana* Colla (B genome). According to Simmonds [1], during their long-term evolution, these two wild species hybridized and interbred, evolving into the modern banana through continuous natural selection and artificial selection. Simmonds and Shepherd [2] developed a banana hybrid identification system using 15 prominent traits, among which 13 traits are related to the reproductive organs and are highly polymorphic between *M. acuminata* and *M. balbisiana*. Based on these phenotypic scoring systems, *M. acuminata* × *M. balbisiana* hybrids can be characterized into different ploidy levels (diploid, triploid, and tetraploid) and several genome groups (e.g., AA, AB, AAA, AAB, and ABB). Generally, one genome group can be divided into several subgroups; for example, the AAA genome group contains the Cavendish, Gros Michel, Red, Lakatan, and Ibota Bota subgroups. According to morphological markers, highly similar cultivated varieties of banana can be classified into subgroups, such as Cavendish banana ‘Brazil’, ‘Williams’, and ‘Pei Chiao’. The identification of banana germplasm is the premise for the evaluation and utilization of banana germplasm resources. However, the management of banana germplasm resources has relied immensely on identification using local names and morphological characters, and the extent of the genetic diversity of banana has not been well established with molecular markers. Most of the morphological markers are polygenic and highly influenced by the environment [3]. Moreover, morphological observation is time-consuming and laborious [4,5,6]. Nevertheless, morphological markers are still used in banana breeding due to the unavailability of other markers [7].

Molecular marker technology has been widely used in germplasm resource identification in recent decades. As described by Kaemmer et al. [8], DNA oligonucleotide and amplification fingerprinting have been successfully employed to detect genetic polymorphisms in 15 representative species and cultivars of the genus *Musa* comprising the AA, AAA, AAAA, AAB, ABB, and BB genotypes. Risterucci et al. [9] demonstrated the usefulness of diversity arrays technology (DArT)—a DNA hybridization-based molecular marker technique that can simultaneously detect variation at numerous genomic loci without sequence information—for genetic diversity analyses of *Musa* genotypes. Numerous studies have utilized a small number of simple sequence repeat (SSR) markers, which have relatively limited genome coverage, in the classification of banana [10,11,12,13]. Several studies analyzed *Musa* genome groups using SSR and amplified fragment length polymorphism (AFLP) markers [14,15,16,17].

Single nucleotide polymorphisms (SNPs), another class of markers, can accurately distinguish highly similar crop germplasm resources [18,19]. Moreover, SNPs have higher genetic stability and are the most promising molecular marker at present for differentiating crop germplasm [18,19]. SNPs are referred to as the third-generation molecular marker [20]. Due to their abundance and genome-wide coverage, and particularly with the advent of high-throughput genotyping methods such as genotyping-by-sequencing, SNP markers have been employed in population genetics studies in banana [21,22,23,24,25]. Cenci et al. [22] used SNP markers in DNA sequencing data related to restriction enzyme sites to study and compare the chromosome structures of 36 banana varieties belonging to the ABB genotype (including different subspecies). Gardoce et al. [26] developed a 1 K SNP genotyping panel that could effectively distinguish between genomic groups based on the filtering of high-quality genome-wide SNPs from the Musa Germplasm Information System and used it to assess the genetic diversity and population structure of 183 *Musa* spp. accessions. Recently, Martin et al. [24] conducted an extensive analysis of whole genome sequencing datasets derived from a comprehensive and varied assembly of both wild and cultivated banana specimens. By pinpointing specific SNP markers, they successfully elucidated the distribution of ancestral genetic components across the banana chromosomes, therefore illuminating novel aspects of the domestication trajectory and the complex evolutionary pathways that have shaped modern cultivars [24]. These studies demonstrate the significant application potential of SNP technology in the identification of banana germplasm resources, suggesting its great promise in this field.

Fragment variation analysis of the internal transcribed spacer (ITS) region in the ribosomal RNA (rRNA) coding gene is widely used to evaluate phylogenetic relationships at a lower taxonomic level because this region has experienced limited natural selection pressure and exhibits great variation, even among closely related species [27,28,29]. ITS is used as a DNA barcoding marker for identifying different species [29,30]. Nwakanma et al. [31] analyzed the ITS fragments of nine banana genotypes (AA, BB, AB, AAA, AAB, ABB, AAAA, AAAB, and AABB) based on restriction fragment length polymorphism (RFLP) and found that the A and B genomes could be distinguished by *Rsa*I digestion. In addition, Dita et al. [32] found that the banana *ACTIN2* gene could be used as a molecular marker to identify the A and B genomes. The above two markers can be used to determine whether banana resources contain A and B genomes, but they are unable to determine the copy number of A and B genomes in polyploids (for example, AAB and ABB cannot be distinguished). Teo et al. [33] used inter-retrotransposon amplified polymorphism (IRAP) to identify the A and B genomes. Subsequently, Nair et al. [34] used copia-IRAP primers to amplify banana germplasm resources, which, together with *Alu*I digestion, could effectively identify AAB and ABB. However, the above methods cannot be used for classification below the genome type. After a thorough analysis of the ITS sequences from 36 banana species (including 42 accessions representing three genera, along with 10 additional ingroup accessions retrieved from the GenBank database and four outgroup accessions), researchers have successfully constructed the phylogeny of the banana family, indicating that ITS can serve as an efficient tool for the identification of wild banana species [4,35,36,37]. In addition, a notable publication emphasizes the potent role of the ITS locus in phylogenetics, particularly in accurately reconstructing relationships within lower taxonomic hierarchies, including groups and subgroups [37]. This previous study has revealed that a substantial proportion of the intraspecific banana hybrids investigated retain unaltered ITS sequences inherited from their respective parent species, underscoring the reliability of the ITS marker in tracing the genomic ancestry of these hybrids [37]. However, the use of ITS sequences for the classification of cultivated banana varieties remains uncertain, and further investigation is warranted.

The National Litchi and Banana Germplasm Resources Garden in China manages and conserves more than 400 accessions of local and introduced cultivars and wild species of *Musa* in the field and through in-vitro conservation. The genetic characterization of the germplasm collection has not been explored extensively at the molecular level. In the present study, it was discovered that the ITS sequencing peaks (especially the 420-bp region) of banana exhibited recognizable and repeatable polymorphism characteristics. Based on this finding, this study developed a new method for identifying banana germplasm resources and successfully divided 62 accessions of banana into 44 ITS types.

## 2. Results

### 2.1. ITS Sequencing of 62 Banana Accessions

The ITS region of banana is composed of two spacer regions and 5.8 S (Supplemental Figure S1). This study utilized two primers, namely ITS L and ITS 4, the positions of which are shown in Supplemental Figure S1, to amplify the ITS fragment of the cultivated variety ‘Brazil’, as described previously by Nwakanma [31]. The ITS sequencing peaks of ‘Brazil’ contained many non-single peaks (Supplemental Figure S2), implying the heterozygosity of ‘Brazil’. This study then tested another line of ‘Brazil’ as a biological repeat. There were few differences among these repetitions, indicating that the ITS peaks were repeatable (Supplemental Figures S2 and S3). Then, the ITS fragments of other Cavendish varieties, namely ‘Pei Chiao’ and ‘Formosana’ (bred from ‘Pei Chiao’), were tested and compared with ‘Brazil’. These lines were not easily distinguishable (Supplemental Figures S2, S4 and S5), implying that these varieties were within the same subgroup of banana according to the ITS results.

By contrast, upon a comparison of the three Cavendish varieties, obvious differences were detected in the Gros Michel banana variety ‘Gros Michel’ and the Ibota Bota banana variety ‘Yangambi KM5’ (Supplemental Figures S2, S6 and S7). Moreover, there was a clear distinction between several AAA group bananas and the Pisang Awak banana (ABB group) variety ‘Guang Fen No.1’ and the hybrid banana variety (ABBB group) ‘Fen Za No.1’ (Supplemental Figures S2 and S6–S9), indicating that ITS could be a suitable marker for genotyping. To explore whether ITS sequencing peaks could reflect the genetic polymorphism of different cultivars, the ITS regions of 62 lines of representative banana germplasm resources were amplified using polymerase chain reaction (PCR) and sequenced, and the peaks were compared.

### 2.2. Classification of Different Banana Subgroups Using the 420-bp Region of ITS

In total, this study examined eight genome groups (AA, AB, AAA, ABB, AAB, ABBB, AAAB, and AAAA), 41 accessions from 17 well-known subgroups (Inarnibal, Sucrier, Pisang Jari Buaya, Ney Pooven, Cavendish, Gros Michel, Red, Lakatan, Ibota Bota, Pisang Awak, Da Jiao, Pelipita, Saba, Pisang Raja, Plantain, Pome, and Silk), 15 accessions of unknown subgroups, and 6 accessions of hybrid banana (Table 1). A segment of the ITS sequence, comprising the first several dozen bases from a selection of bananas, is relatively homozygous; we performed sequence alignments on these segments. We found that the number of SNP sites in this ITS region among these bananas is very limited (Supplemental Figure S10), indicating the necessity of analyzing ITS sequencing peaks. The analysis of 62 sequencing maps proved challenging. Hence, a 420-bp region was selected as a representative target for streamlining comparison processes due to its indicative polymorphic features (Supplemental Figures S2–S9). This region constitutes a 20-bp window near the 420-bp position on the ITS sequencing map, exhibiting either the motif ‘NNNCCCCCNNGGGGGNNNNN’ in all samples except No.50 or the distinct pattern ‘CCCCCCNNNNNNNNNNNNNN’ uniquely found in No.50. It was evident that the 420-bp region of many cultivars differed. The ITS sequences of only four banana varieties (Morong Princesa, Rose, Zhong Jiao No.9, and FHIA-01) were relatively homozygous (Supplemental Figures S11–S14). The alignment of the ITS sequences from these four banana cultivars with those of seven wild banana species revealed that the initial portion of the 420-bp region was highly conserved (TGCCCCCTCGGGG), whereas the latter part exhibited greater variability (Supplemental Figure S15). To facilitate reference, recording, and comparison, different combinations of letters (indicating the genome group) and numbers were used to represent different polymorphism types of the ITS 420-bp region.

**Figure 1:**
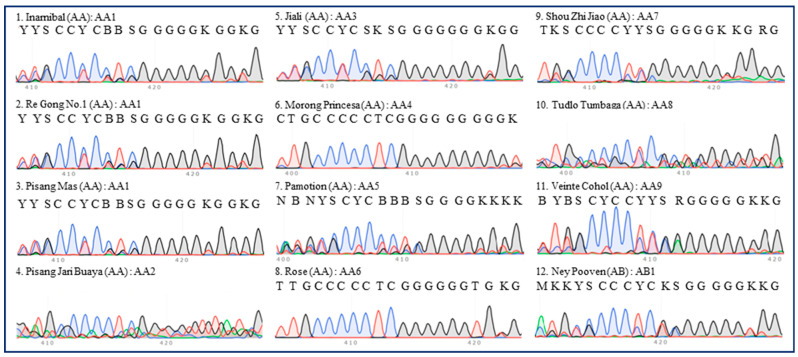
**ITS sequence peaks (420-bp region) of 12 accessions of diploid banana.** Eleven accessions of AA group banana (‘Inarnibal’, ‘Re Gong No.1’, ‘Pisang Mas’, ‘Pisang Jari Buaya’, ‘Jia Li’, ‘Morong Princesa’, ‘Pamotion’, ‘Rose’, ‘Shou Zhi Jiao’, ‘Tudlo Tumbaga’, and ‘Veinte Cohol’) and one accession (‘Ney Pooven’) of AB group banana were tested. The serial number, name, group, and ITS type of each accession are indicated in each panel. Most of the regions are labeled in accordance with the IUPAC nucleotide code for ambiguous nucleobases.

There were nine ITS types (AA1–9) among 11 accessions of AA group bananas (Table 1). While two Inarnibal bananas (‘Inarnibal’ and ‘Re Gong No.1’) and Pisang Mas banana (‘Pisang Mas’) were classified into one ITS type (AA1), another eight accessions from different subgroups represented eight ITS types (AA2–9) (Figure 1). The ITS 420-bp region of the AB group Ney Pooven was an exception and was referred to as AB1 (Figure 1).

**Figure 2:**
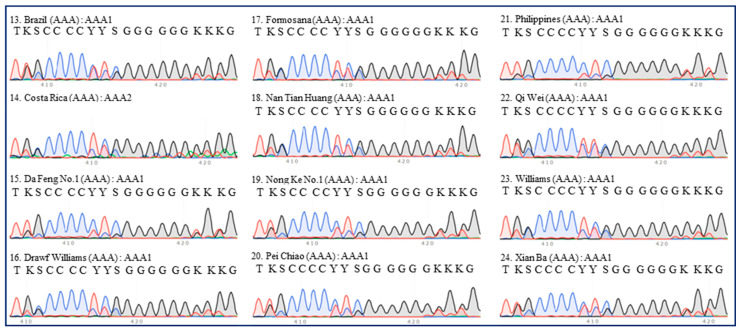
**ITS sequence peaks (420-bp region) of 12 accessions of Cavendish banana.** Twelve accessions of Cavendish (‘Brazil’, ‘Costa Rica’, ‘Da Feng No.1’, ‘Dwarf Williams’, ‘Formosana’, ‘Nan Tian Huang’, ‘Nong Ke No.1’, ‘Pei Chiao’, ‘Philippines’, ‘Qi Wei’, ‘Williams’, and ‘Xian Ba’) were tested. The serial number, name, group, and ITS type of each accession are indicated in each panel. Most of the regions are labeled in accordance with the IUPAC nucleotide code for ambiguous nucleobases.

**Figure 3:**
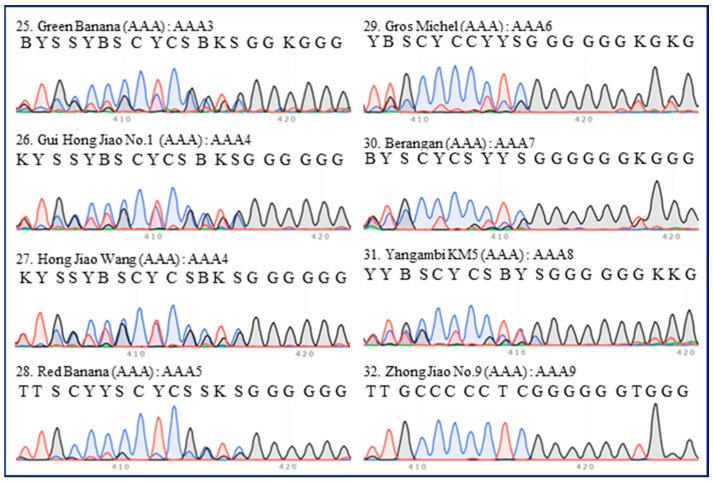
**ITS sequence peaks (420-bp region) of eight accessions of AAA group banana, excluding Cavendish.** Four accessions of Red (‘Green Banana’, ‘Gui Hong Jiao No.1’, ‘Hong Jiao Wang’, and ‘Red Banana’), one accession of Gros Michel (‘Gros Michel’), one accession of Lakatan (‘Berangan’), one accession of Ibota Bota (‘Yangambi KM5’), and one accession of hybrid banana (‘Zhong Jiao No.9’) were tested. The serial number, name, group, and ITS type of each accession are indicated in each panel. All regions are labeled in accordance with the IUPAC nucleotide code for ambiguous nucleobases.

There were nine ITS types (AAA1–9) among 20 accessions of AAA group bananas (Table 1). All Cavendish bananas were included in the same ITS type (AAA1), except ‘Costa Rica’ (Figure 2). Some extra peaks were observed in the ITS 420-bp region of ‘Costa Rica’, which was thus referred to as the AAA2 type, despite its similarity to the 420-bp region of the other 11 Cavendish accessions (Figure 2). Four red banana accessions were clustered into three types (AAA3–5), and these types were evidently similar (Figure 3). ‘Gros Michel’ (Gros Michel subgroup) belonged to type AAA6 (Figure 3). ‘Berangan’ (Lakatan subgroup), ‘Yangambi KM5’ (Ibota Bota subgroup), and ‘Zhong Jiao No.9’ (‘FHIA-01’ × ‘SH-3142’) were types AAA7, AAA8, and AAA9, respectively (Figure 3).

**Figure 4:**
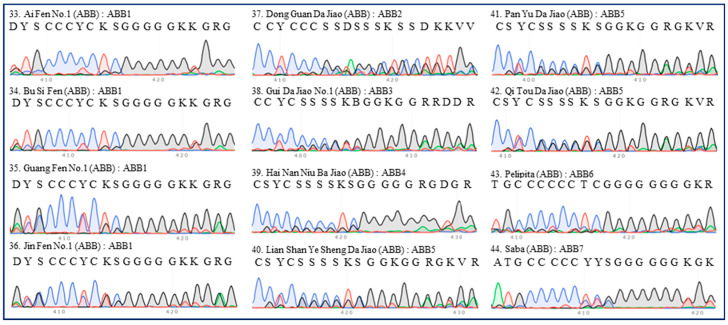
**ITS sequence peaks (420-bp region) of 12 accessions of ABB group banana.** Four accessions of Pisang Awak (‘Ai Fen No.1’, ‘Bu Si Fen’, ‘Guang Fen No.1’, and ‘Jin Fen No.1’), six accessions of Da Jiao (‘Dong Guan Da Jiao’, ‘Gui Da Jiao No.1’, ‘Hai Nan Niu Ba Jiao’, ‘Lian Shan Ye Sheng Da Jiao’, ‘Pan Yu Da Jiao’, and ‘Qi Tou Da Jiao’), one accession of Pelipita (‘Pelipita’), and one accession of Saba (‘Saba’) were tested. The serial number, name, group, and ITS type of each accession are indicated in each panel. All regions are labeled in accordance with the IUPAC nucleotide code for ambiguous nucleobases.

**Figure 5:**
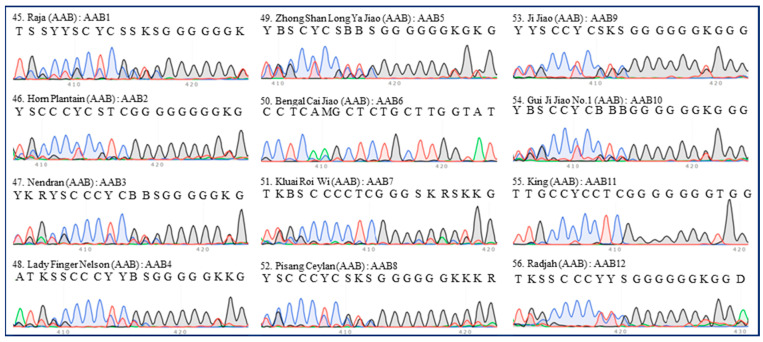
**ITS sequence peaks (420-bp region) of 12 accessions of AAB group banana.** One accession of Pisang Raja (‘Raja’), two accessions of Plantain (‘Horn Plantain’ and ‘Nendran’), one accession of Pome (‘Lady Finger Nelson’), one accession of Silk (‘Zhong Shan Long Ya Jiao’), and seven accessions of unknown subgroups (‘Bengal Cai Jiao’, ‘Kluai Roi wi’, ‘Pisang Ceylan’, ‘Ji Jiao’, ‘Gui Ji Jiao No.1’, ‘King’, and ‘Radjah’) were tested. The serial number, name, group, and ITS type of each accession are indicated in each panel. All regions are labeled in accordance with the IUPAC nucleotide code for ambiguous nucleobases.

**Figure 6:**
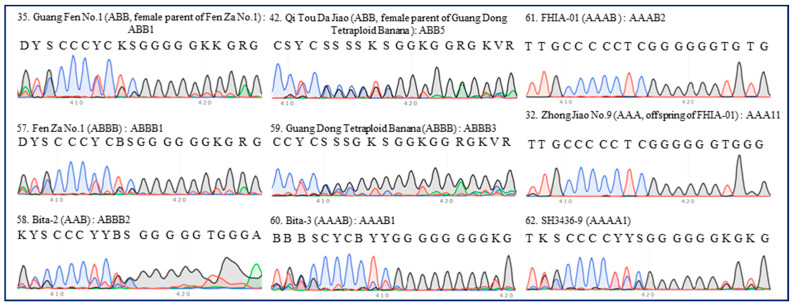
**ITS sequence peaks (420-bp region) of several accessions of tetraploid banana and their related cultivars.** Seven accessions of tetraploid banana (‘Fen Za No.1’, ‘Bita-2’, ‘Guang Dong Tetraploid Banana’, ‘Bita-3’, ‘FHIA-01’, ‘Zhong Jiao No.9’, and ‘SH3436-9’) were tested. The results for ‘Guang Fen No.1’ (female parent of ‘Fen Za No.1’), ‘Qi Tou Da Jiao’ (female parent of ‘Guang Dong Tetraploid Banana’), and ‘Zhong Jiao No.9’ (offspring of ‘FHIA-01’) are shown for comparison. The serial number, name, group, and ITS type of each accession are indicated in each panel. All regions are labeled in accordance with the IUPAC nucleotide code for ambiguous nucleobases.

**Figure 7:**
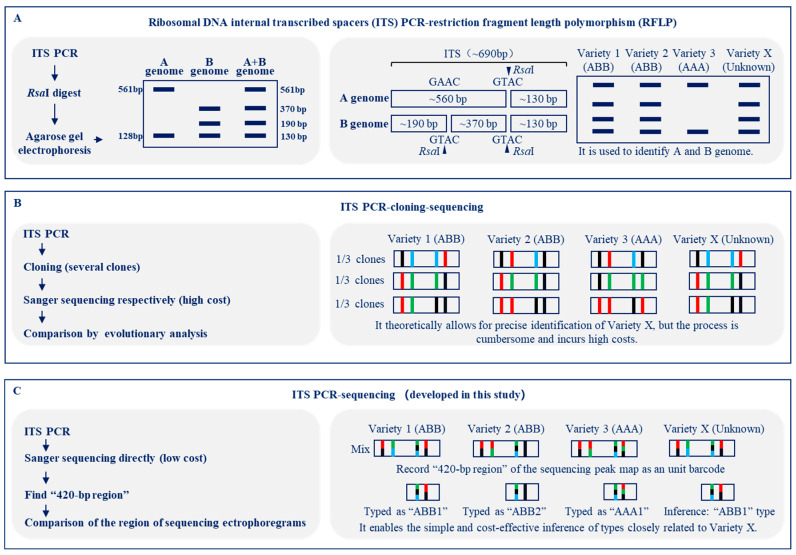
**Schematic diagram elucidating the steps and principles of banana identification using ITS sequences.** As an illustration, suppose there is a need to roughly distinguish four banana samples: three known cultivars labeled ‘Variety 1 (ABB)’, ‘Variety 2 (ABB)’, and ‘Variety 3 (AAA)’, alongside an unknown variety designated ‘Variety X’ (known to be one of Varieties 1–3). (**A**) For ITS PCR-restriction fragment length polymorphism (RFLP) analysis, the ITS PCR product is digested with the restriction enzyme *Rsa*I, followed by agarose gel electrophoresis [31]. (**B**) For ITS PCR-cloning-sequencing, the ITS PCR product is ligated into a vector, and Sanger sequencing was performed on several individual bacterial colonies separately [37]. (**C**) For ITS PCR sequencing, the ITS PCR product is directly subjected to Sanger sequencing, with subsequent comparisons made of the variations at specific positions in the Sanger sequencing ectrophoregrams.

There were seven ITS types (ABB1–7) among 12 accessions of ABB group bananas (Table 1). All four Pisang Awak bananas (‘Ai Fen No.1’, ‘Bu Si Fen’, ‘Guang Fen No.1’, and ‘Jin Fen No.1’) were clustered into one type (ABB1) (Figure 4). Six Da Jiao (‘Dong Guan Da Jiao’, ‘Gui Da Jiao No.1’, ‘Hai Nan Niu Ba Jiao’, ‘Lian Shan Ye Sheng Da Jiao’, ‘Pan Yu Da Jiao’, and ‘Qi Tou Da Jiao’) were clustered into four types (ABB2–5), though they did exhibit similarities (Figure 4).

Interestingly, none of the 12 accessions of AAB group bananas shared the same ITS type as the others (Table 1). Even the two plantains (‘Horn Plantain’ and ‘Nendran’) differed (typed as AAB2 and AAB3, respectively) (Figure 5). The types of ‘Ji Jiao’ and ‘Gui Ji Jiao No.1’, which should have been the same type of cultivar, were similar (Figure 5).

### 2.3. Identification of Hybrid Bananas Using the 420-bp Region of ITS

Six accessions of tetraploid banana (including the ABBB, AAAB, and AAAA groups) were hybrid bananas, representing six ITS types (ABBB1–3, AAAB1–2, and AAAA1) (Table 1). Despite the ITS 420-bp region of ‘Fen Za No.1’ (typed as ABBB1) being similar to that of ‘Guang Fen No.1’ (ABB1), a Pisang Awak banana that is the female parent of ‘Fen Za No.1’, it was easy to differentiate between types ABBB1 and ABB1 (Figure 6). This implied that ITS sequencing could be applied in identifying banana hybrid offspring. In line with expectations, a tiny discrepancy was found between the ITS 420-bp region of ‘Guang Dong Tetraploid Banana’ (typed as ABBB3) and its female parent ‘Qi Tou Da Jiao’ (ABB5), and ‘Zhong Jiao No.9’ (typed as AAA11) and its female parent ‘FHIA-01’ (typed as AAAB2) (Figure 6).

## 3. Discussion

The genetic background of modern cultivated banana is complex. This is primarily because (1) during evolution, the ancestors of cultivated banana were formed by different wild species and interspecific hybridization, which endowed them with rich genetic diversity; and (2) similarly, the long-term vegetative reproduction of cultivated banana varieties resulted in the accumulation of significant genetic diversity [38,39]. Therefore, the differences in ITS sequences in cultivated banana can reflect the differences in cultivation types to some extent [37]. 

In the present analysis, the superposition results of the SNPs and insertion/deletion sets from the genomes of banana in the first-generation sequencing peak map of the ITS fragment were used as a fingerprint of an ITS polymorphism. The experiments in the present study confirmed that the ITS sequencing peaks, and particularly the 420-bp region, of different banana cultivars, could accurately reflect their genetic background to some extent. Using the 420-bp region as a marker, 62 accessions of banana were clustered into 44 types. In the ITS of several wild and cultivated banana species, the first half of the 420-bp region (TGCCCCCTCGGGG) is highly conserved (Supplemental Figure S15), whereas the second half exhibits clear insertion or deletion sites, making it easily identifiable and suitable for comparative purposes. Moreover, since the ITS mixture is directly sequenced, any insertions or deletions occurring in the sequence preceding the 420-bp region (including ITS1) can lead to frameshifts that affect the 420-bp region. Consequently, the 420-bp region effectively captures a composite picture of variations in ITS. For efficient classification of numerous samples, detailed full-sequence electropherogram comparisons are too cumbersome and impractical. We suggest focusing on the 420-bp region for genotyping. If required, full-sequence electropherogram analyses for samples with matching ITS types can follow, notably lightening the workload. The improvement of banana cultivars through crossbreeding is promising [40]. The method developed in this work appears to be effective for identifying the offspring and, therefore, will be useful for the early detection of hybrid banana.

Earlier reports documented three distinct techniques utilizing ITS for the differentiation of banana varieties: ITS PCR-RFLP [31], ITS PCR followed by cloning and sequencing [37], and ITS extraction from genome second-generation sequencing [37]. In the ITS PCR-RFLP analysis, the resultant ITS PCR product undergoes digestion with the restriction enzyme *Rsa*I, followed by agarose gel electrophoresis to assess the polymorphic profiles of the restriction fragments (Figure 7A) [31]. This method has limitations in precisely categorizing genomic types or distinguishing varieties that share an identical genomic composition (Figure 7A) [31]. Alternatively, the ITS PCR-cloning-sequencing approach entails the ligation of the PCR product into a plasmid vector, followed by the separate Sanger sequencing of 15–85 individual bacterial clones, with subsequent polymorphism analyses among the sequenced data (Figure 7B) [37]. This method allows sequencing a single copy of ITS from the genome and avoids overlapping peaks in the chromatogram [37]. Although capable of theoretically delivering precise varietal identifications, this technique is encumbered by a tedious process and substantial costs (Figure 7B) [37]. Despite its cost comparability to whole genome resequencing, this method yields information from a single locus, limiting its practical utility [37]. Genotyping approaches that depend on high-throughput detection technologies, such as DArT, EcoTILLING, and whole genome sequencing, are undeniably the most precise [9,23,24,37]. Nevertheless, the primary disadvantages of these technologies are their high costs, extended time requirements, and the complexity of data analysis, which collectively hinder their widespread adoption for routine research and sample testing in an industrial context [9,23,24,37,41]. 

DNA barcoding has emerged as a potent biotechnological advantage, hinging upon the employment of 400–800 base pair-long, standardized, unique DNA sequences derived from mitochondria (such as CO1), plastids (such as *rbc*L), or the nucleus (ITS), enabling precise species identification and classification [30,42]. Essentially, ITS PCR sequencing constitutes a DNA barcoding methodology [30,42]. ITS PCR sequencing streamlines this process: the ITS PCR product is directly subjected to Sanger sequencing, with a focus on examining variations at a pivotal region of interest, notably the 420-bp region, via scrutiny of the sequencing chromatograms (Figure 7C). This strategy offers a simplified and cost-efficient means to infer types closely affiliated with the unidentified variety, therefore enhancing the practicality and efficiency of the analytical process. 

Mislabeled accessions and duplicate sampling are frequent issues encountered in germplasm repositories [43]. As an example, imagine a scenario in which there is a requirement to roughly distinguish between four banana samples: three known cultivars labeled ‘Variety 1 (ABB)’, ‘Variety 2 (ABB)’, and ‘Variety 3 (AAA)’, alongside an unknown variety designated ‘Variety X’ (known to be one of Varieties 1–3) (Figure 7). Conducting ITS PCR-cloning-sequencing or whole genome sequencing for these samples in China would entail a minimum expense of approximately USD 80 per sample and turnaround times of about one month and 5 days, respectively. These processes would provide comprehensive sequencing data, enabling detailed comparative analyses crucial for accurately identifying and characterizing the genetic differences among the samples, including the unknown ‘Variety X’ [37]. In comparison, distinguishing between these samples would be easy to achieve by employing the approach developed in this paper (Figure 7), which requires only approximately USD 2 per sample and a single day without necessitating intricate bioinformatics analysis. ITS PCR sequencing, while seemingly less sophisticated, offers practicality by effectively distinguishing these three banana types and presents a lower barrier to entry, making it more accessible to individuals without a bioinformatics background. 

Although a variety of tools can be used [3], the classification of banana remains a challenge. For example, the classification of cultivated ABB is necessary because the more popular names (Saba, Pisang Awak, Peyan, Bluggoe, and Monthan) represent a cluster of closely related cultivars generated by somatic variation [22]. The nomenclature of the entire ABB group is difficult to resolve, given that the only source of information is the local name of each variant in Asia [22]. This difficulty was confirmed by Saraswathi [44], who combined morpho-taxonomic descriptors and SSR markers and attempted to discriminate the Indian subgroups. Using DArT and SSR markers on a wider sample, researchers confirmed that the classification was consistent for accessions belonging to the subgroups Pelipita, Klue Teparod, and Pisang Awak [21,45]. However, Sardos [35] found that accessions classified as belonging to the subgroups Saba, Monthan, Bluggoe, Ney Mannan, or Peyan were often misclassified. In this paper, using ITS sequencing, we revealed that the classification of most accessions was in accordance with classification information obtained from the Musa Germplasm Information System (https://www.crop-diversity.org/mgis/, accessed on 20 November 2023) and the National Horticultural Germplasm Resources Center of China (http://www.nhgrc.cn/pcindex/, accessed on 20 November 2023). For example, 11 of the 12 Cavendish bananas used in this study were clustered into one ITS type (Figure 2), and all four Pisang Awak bananas were clustered into one ITS type (Figure 4). There was an exception. Two different subgroups, Inarnibal and Sucrier, were clustered into one ITS type (Figure 2), suggesting that supplementary examination via alternative technologies (such as SSR) is warranted when distinguishing between these two subgroups. 

To date, SSR has been the primary molecular identification method for bananas [10,11,12,13]. In the global *Musa* gene bank (International Transit Centre, Leuven, Belgium), the SSR profiling of banana samples is conducted systematically through various rounds of batch processing on a regular basis [43]. The SSR analysis process initially entails designing and screening for the most suitable primer pairs (ranging from several to dozens) that can effectively discriminate among samples in the repository, a step that incurs both considerable time and financial costs that cannot be overlooked [11,13,20,46]. To ensure reliable and consistent results from SSR analysis, the use of capillary electrophoresis equipment and costly assay kits, often involving batch testing, is typically indispensable [20,43]. While the experimental procedures and data analysis involved in SSR are not exceedingly complicated, they do necessitate a level of specialized training [20,43]. These characteristics collectively restrict the application of SSR, especially for researchers who do not have prior experience with SSR methodologies. In contrast to SSR, ITS PCR sequencing only requires amplification with a fixed pair of primers, followed by a single Sanger sequencing reaction of the PCR product. The process is straightforward and manageable, eliminating the need for batch testing. Results obtained at different times or locations are easily comparable. Although ITS PCR sequencing involves outsourcing to sequencing companies, Sanger sequencing services are well established and come at an affordable cost in many areas of the world.

The method outlined in this study can effectively improve the efficiency of banana germplasm identification as an auxiliary means of character identification and genome type identification. A simple and reliable genotyping method for banana clones, hybrids, species, and relatives will facilitate germplasm management and support breeding initiatives toward a marker-based approach. ITS sequencing might also be useful in the identification of other crops. Using this method, further sequencing peak maps of other sequences can be mined and developed as molecular markers for germplasm identification. 

Although utilizing mixed peaks from Sanger sequencing for genotyping is a commonly adopted practice in applications such as mutant identification post-genome editing or banana classification [47,48], the principal limitation of our approach is the recurrent presence of sequencing peaks characterized by weak signals. To address this issue maximally, quantitative comparison of sequencing peaks was avoided, with the focus shifted to qualitative analysis, therefore minimizing Sanger sequencing errors. Differences between genotypes based on ITS Sanger chromatograms could be categorized into ‘major discrepancies’ and ‘minor variations’. ‘Major discrepancies’ involve distinctly different peak patterns, easily discernible, while ‘minor variations’ refer to faint, less definitive peaks whose authenticity is harder to ascertain. Both types can be confirmed through replication; inconsistencies across replicates lead to disregard of ‘minor variations’, yet consistently observed clear peaks (‘major discrepancies’) remain valid criteria. We acknowledge room for improvement in the precision of ITS PCR sequencing, and future endeavors aim to employ cost-effective next-generation sequencing to unravel single-copy ITS sequences. 

## 4. Material and Methods

### 4.1. Materials

The banana germplasm used in this paper was obtained from the National Litchi and Banana Germplasm Resources Garden. As most edible cultivars are derived from *M. acuminata* (AA genome) and *M. balbisiana* (BB genome), the study was restricted to accessions of these two species and those derived from them. Two biological repeats of each germplasm were tested.

### 4.2. Rapid Extraction of Banana Genomic DNA

DNA extraction was performed as described in Dellaporta et al. [49] with minor modifications. Briefly, a small piece of banana leaf was ground and treated with DNA extraction solution, followed by incubation, centrifugation, and the transfer of the supernatant to a new tube. Equal volumes of isopropanol were added, mixed, and centrifuged, and the supernatant was discarded. The remaining pellet underwent ethanol washes, drying, rehydration in sterile water, and storage at −20 °C. During PCR setup, 1–2 μL of the resulting DNA solution was utilized.

### 4.3. PCR, Sequencing, and Analysis

The banana ITS region was amplified using the ITSL (TCGTAACAAGGTTTCCGTAGGTG) and ITS4 (TCCTCCGCTTATTGATATGC) primers [50,51]. The PCR reaction conditions included pre-denaturation at 95 °C for 5 min, denaturation for 30 s, annealing at 55 °C for 30 s, extension at 72 °C for 30 cycles, and extension for 10 min. Following the PCR reaction, the product was sent to a sequencing company (Beijing Tsingke Biotech Co., Ltd., Beijing, China) for sequencing with the ITSL primers (the ITS PCR product was about 700 bp in size). For each DNA template, PCR and sequencing were repeated at least twice. After obtaining the sequencing results, the Snap Gene 2.3.2 software was used to open the .ab1 file and check the sequencing peak diagram. If there was visible sample pollution, an abnormal peak, or an insufficient sequencing length, the sample was retested. Regions of the ITS sequencing peak map, specifically a 20-bp window around the 420-bp position, revealed distinct motifs: ‘NNNCCCCCNNGGGGGNNNNN’ was consistently observed across all samples except No.50 (Bengal Cai Jiao), whereas ‘CCCCCCNNNNNNNNNNNNNN’ was unique to No.50. Screenshots of these regions were captured for comparative analysis. Here, ‘N’ denotes any nucleotide, and ‘C’ and ‘G’ denote combinations of bases that include ‘Cytosine’ and ‘Guanine’, respectively. To ensure the stability and reliability of the results, the results of different biological repetitions and technical repetitions were compared for each banana germplasm resource to ensure consistency among multiple repetitions. The ITS 420-bp regions are labeled in accordance with the IUPAC nucleotide code for ambiguous nucleobases, except for which the signal is too complex to be reliably recorded. This method avoids quantitative peak comparisons in sequencing, concentrating on qualitative analysis (i.e., presence or absence of peaks), thus reducing Sanger sequencing-related errors. Our recommendation is to prioritize the use of the 420-bp region for genotyping. Should the need arise, conducting full-sequence ectrophoregrams comparisons for samples with identical ITS types can be done subsequently.

## Figures and Tables

**Table 1:** Information on the 62 banana accessions.

Serial No.	Name	Accession No.	Group	Subgroup	ITS Type
1	Inarnibal	ITC0477	AA	Inarnibal	AA1
2	Re Gong No.1	-	AA	Inarnibal	AA1
3	Pisang Mas	ITC1403	AA	Sucrier	AA1
4	Pisang Jari Buaya	ITC0312	AA	Pisang Jari Buaya	AA2
5	Jia Li (mutant of Kluai Lep Mu Nang)	-	AA	Unknown	AA3
6	Morong Princesa	ITC1150	AA	Unknown	AA4
7	Pamotion	-	AA	Unknown	AA5
8	Rose	ITC0712	AA	Unknown	AA6
9	Shou Zhi Jiao	-	AA	Unknown	AA7
10	Tudlo Tumbaga	ITC1231	AA	Unknown	AA8
11	Veinte Cohol	ITC1031	AA	Unknown	AA9
12	Ney Pooven	ITC0459	AB	Ney Pooven	AB1
13	Brazil	-	AAA	Cavendish	AAA1
14	Costa Rica	-	AAA	Cavendish	AAA2
15	Da Feng No.1	-	AAA	Cavendish	AAA1
16	Dwarf Williams	-	AAA	Cavendish	AAA1
17	Formosana	ITC1597	AAA	Cavendish	AAA1
18	Nan Tian Huang	-	AAA	Cavendish	AAA1
19	Nong Ke No.1	-	AAA	Cavendish	AAA1
20	Pei Chiao	-	AAA	Cavendish	AAA1
21	Philippines	-	AAA	Cavendish	AAA1
22	Qi Wei	-	AAA	Cavendish	AAA1
23	Williams	ITC0570	AAA	Cavendish	AAA1
24	Xian Ba	-	AAA	Cavendish	AAA1
25	Green Banana	-	AAA	Red	AAA3
26	Gui Hong Jiao No.1	-	AAA	Red	AAA4
27	Hong Jiao Wang	-	AAA	Red	AAA4
28	Red Banana	-	AAA	Red	AAA5
29	Gros Michel	ITC1122	AAA	Gros Michel	AAA6
30	Berangan	ITC1287	AAA	Lakatan	AAA7
31	Yangambi KM5	-	AAA	Ibota Bota	AAA8
32	Zhong Jiao No.9	-	AAA	FHIA-01 × SH-3142	AAA9
33	Ai Fen No.1	-	ABB	Pisang Awak	ABB1
34	Bu Si Fen	-	ABB	Pisang Awak	ABB1
35	Guang Fen No.1	-	ABB	Pisang Awak	ABB1
36	Jin Fen No.1	-	ABB	Pisang Awak	ABB1
37	Dong Guan Da Jiao	-	ABB	Da Jiao	ABB2
38	Gui Da Jiao No.1	-	ABB	Da Jiao	ABB3
39	Hai Nan Niu Ba Jiao	-	ABB	Da Jiao	ABB4
40	Lian Shan Ye Sheng Da Jiao	-	ABB	Da Jiao	ABB5
41	Pan Yu Da Jiao	-	ABB	Da Jiao	ABB5
42	Qi Tou Da Jiao	-	ABB	Da Jiao	ABB5
43	Pelipita	ITC0396	ABB	Pelipita	ABB6
44	Saba	ITC1138	ABB	Saba	ABB7
45	Raja	-	AAB	Pisang Raja	AAB1
46	Horn Plantain	-	AAB	Plantain	AAB2
47	Nendran	-	AAB	Plantain	AAB3
48	Lady Finger Nelson	ITC0582	AAB	Pome	AAB4
49	Zhong Shan Long Ya Jiao	-	AAB	Silk	AAB5
50	Bengal Cai Jiao	-	AAB	Unknown	AAB6
51	Kluai Roi wi	-	AAB	Unknown	AAB7
52	Pisang Ceylan	ITC1441	AAB	Unknown	AAB8
53	Ji Jiao	-	AAB	Unknown	AAB9
54	Gui Ji Jiao No.1	-	AAB	Unknown	AAB10
55	King	-	AAB	Unknown	AAB11
56	Radjah	-	AAB	Unknown	AAB12
57	Fen Za No.1	-	ABBB	Guang Fen No.1 × Musa Balbisiana	ABBB1
58	Bita-2	ITC1296	ABBB	Fougamou (AAB, Pisang Awak) × Musa Balbisiana 1-63	ABBB2
59	Guang Dong Tetraploid Banana	-	ABBB	Qi Tou Da Jiao×BB	ABBB3
60	Bita-3	ITC1297	AAAB	Laknau (AAB, Laknau) × Tjau Lagada (AA)	AAAB1
61	FHIA-01	ITC0504	AAAB	Prata Ana × SH-3142	AAAB2
62	SH3436-9	ITC1283	AAAA	Unknown	AAAA1

Note: Details on the classification of banana groups and subgroups were mainly obtained from the Musa Germplasm Information System (https://www.crop-diversity.org/mgis/, accessed on 20 November 2023) and the National Horticultural Germplasm Resources Center of China (http://www.nhgrc.cn/pcindex/, accessed on 20 November 2023).

## Data Availability

The original contributions presented in the study are included in the article/Supplementary Material, further inquiries can be directed to the corresponding author.

## Supplementary Materials

The following supporting information can be downloaded at: https://www.mdpi.com/article/10.3390/plants13162173/s1, Figure S1: Banana ribosomal DNA unit structure. Figure S2: ITS sequencing peak map of one clone of the Cavendish banana Brazil variety. Figure S3: ITS sequencing peak map of another clone of the Cavendish banana Brazil variety. Figure S4: ITS sequencing peak map of the Cavendish banana Pei Tiao variety. Figure S5: ITS sequencing peak map of the Cavendish banana Formosana variety. Figure S6: ITS sequencing peak map of the Gros Michel banana Gros Michel variety. Figure S7: ITS sequencing peak map of the Ibota Bota banana Yangambi KM5 variety. Figure S8: ITS sequencing peak map of the Pisang Awak banana Guang Fen No.1 variety. Figure S9: ITS sequencing peak map of the hybrid banana Fen Za No.1 variety. Figure S10: Sequence alignment of the partial ITS regions across 46 accessions of banana. Figure S11: ITS sequencing peak map of the AA banana Morong Princesa variety. Figure S12: ITS sequencing peak map of the AA banana Rose variety. Figure S13: ITS sequencing peak map of the hybrid banana Zhong Jiao No.9 variety. Figure S14: ITS sequencing peak map of the hybrid banana FHIA-01 variety. Figure S15: Sequence alignment of the ITS region from 7 wild banana species and 4 cultivated banana varieties.
