# Supplementary material for: Banana Classification Using Sanger Sequencing of the Ribosomal DNA Internal Transcribed Spacer (ITS) Region
Source: Plants (Basel). 2024 Aug 6;13(16):2173. doi: 10.3390/plants13162173 (PMC11359176; doi:10.3390/plants13162173)
Supplement: Supplementary file 1 [file plants-13-02173-s001.zip › plants-3039536-supplementary.pdf]

Supplementary material

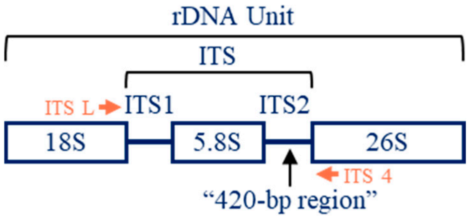

Supplemental Figure S1. Banana ribosomal DNA unit structure. The ribosomal DNA unit (rDNA) of banana containing 18S, ITS1, 5.8S, ITS2, and 26S. Internal transcribed spacers (ITS), including ITS1, 5.8S, and ITS2, was cloned using one pair of primers (ITS L and ITS 4, the positions are indicated) in this study. The "420-bp region" is located between 5.8S and 26S.

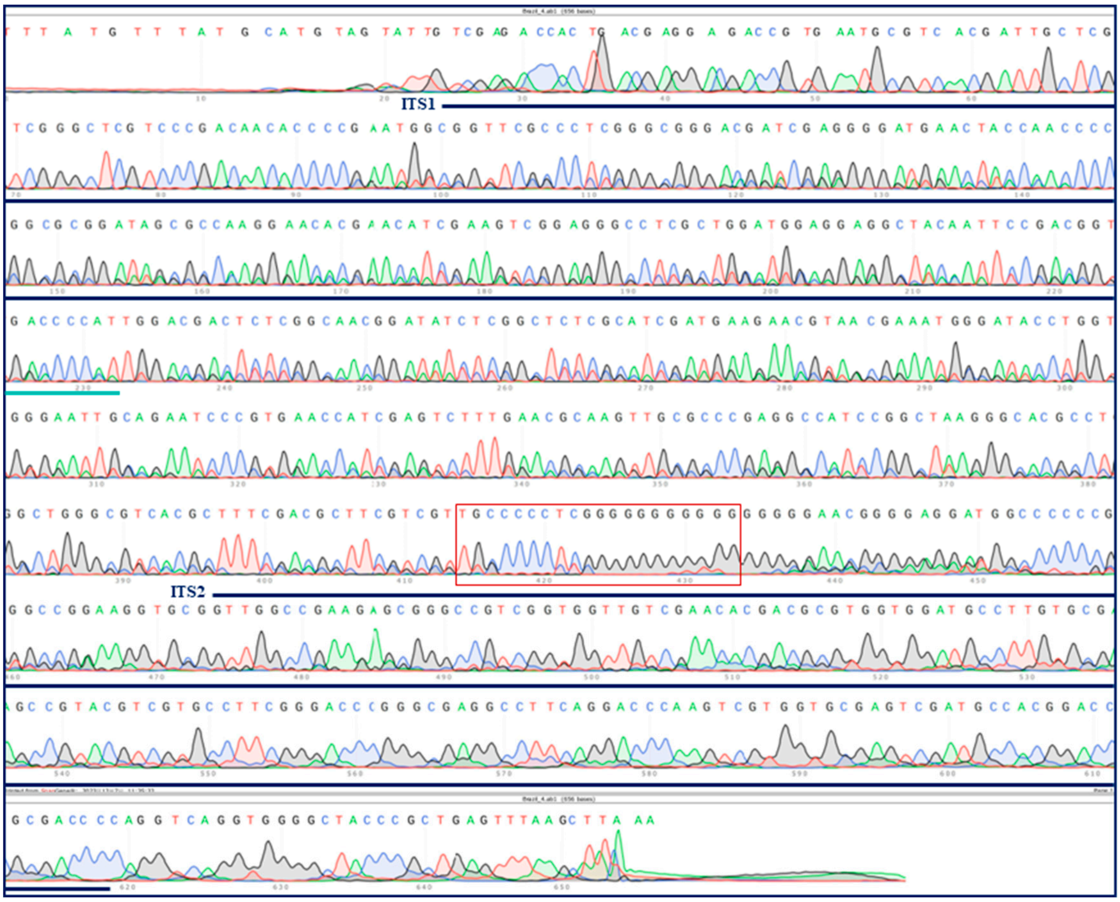

Supplemental Figure S2. ITS sequencing peak map of one clone of the Cavendish banana Brazil variety. The ITS of another biological repetition of 'Brazil' was amplified by PCR and sequenced through Sanger sequencing. The positions of ITS1, ITS2 and the "420-bp region" are indicated.

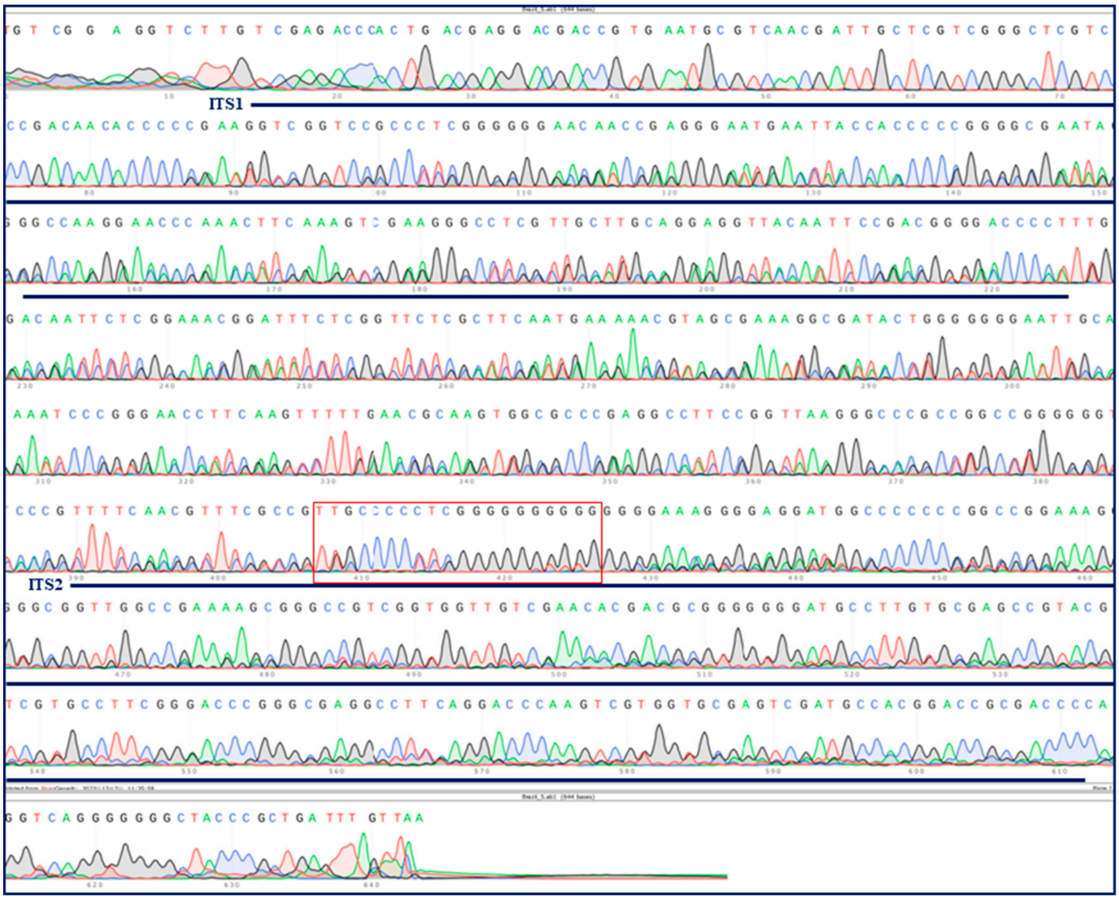

Supplemental Figure S3. ITS sequencing peak map of another clone of the Cavendish banana Brazil variety. ITS of another ‘Brazil’ were amplified by PCR and sequenced through Sanger sequencing. The positions of ITS1, ITS2 and the “420-bp region” are indicated.

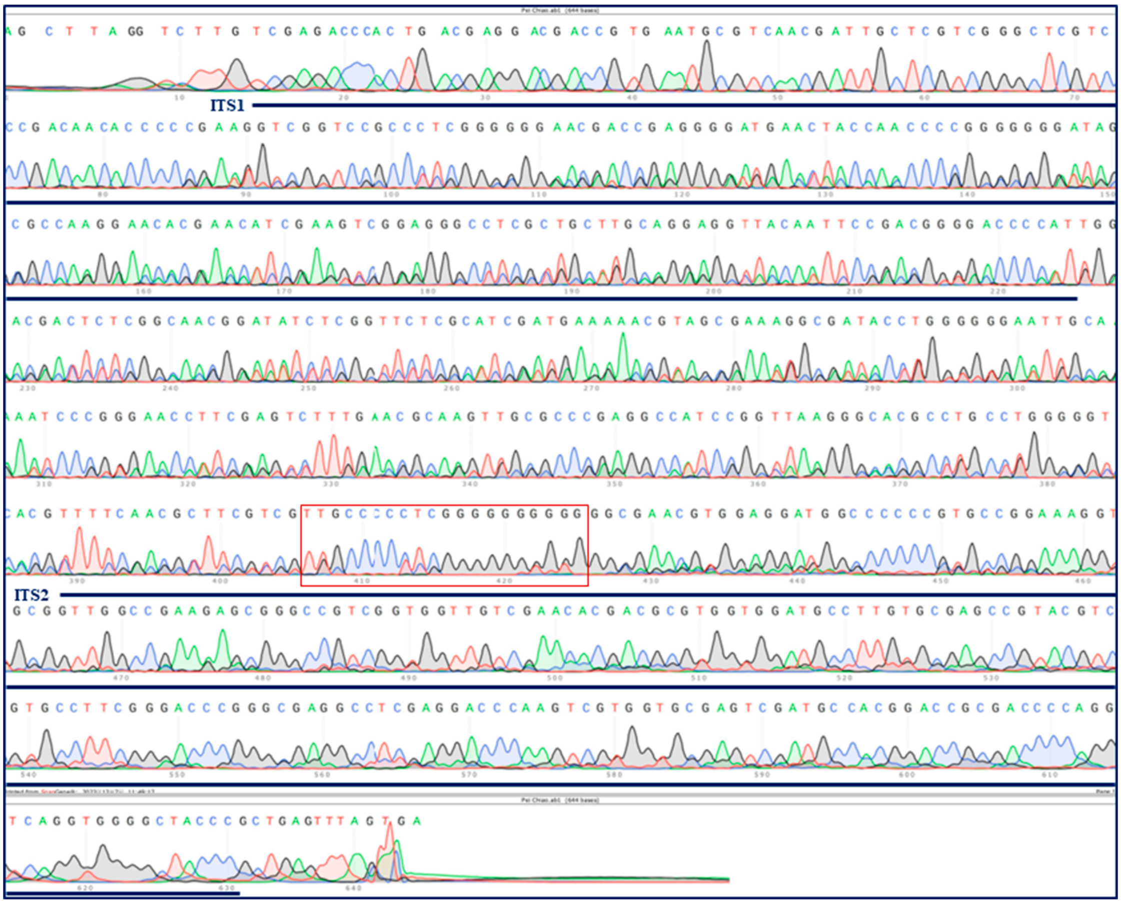

Supplemental Figure S4. ITS sequencing peak map of the Cavendish banana Pei Tiao variety. ITS of ‘Pei Chiao’ were amplified by PCR and sequenced through Sanger sequencing. The positions of ITS1, ITS2 and the “420-bp region” are indicated.

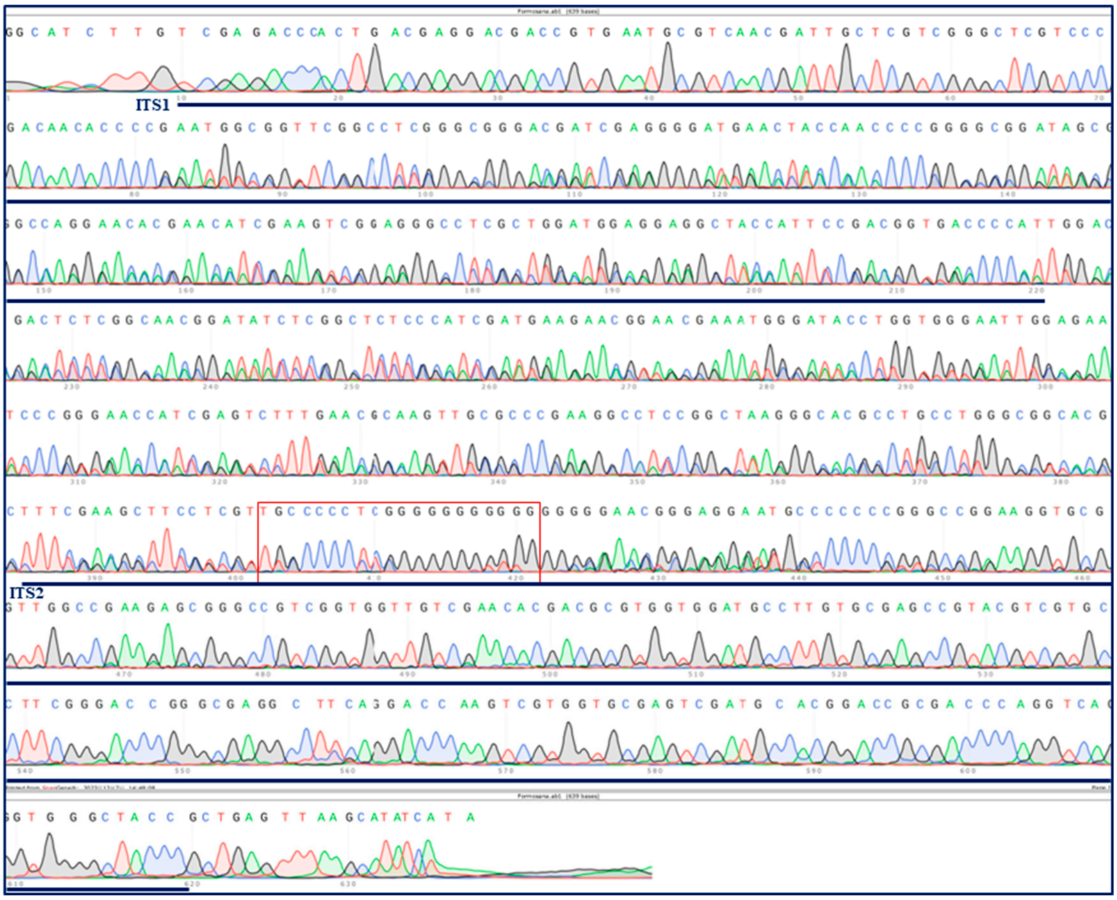

Supplemental Figure S5. ITS sequencing peak map of the Cavendish banana Formosana variety. ITS of 'Formosana' were amplified by PCR and sequenced through Sanger sequencing. The positions of ITS1, ITS2 and the "420-bp region" are indicated.

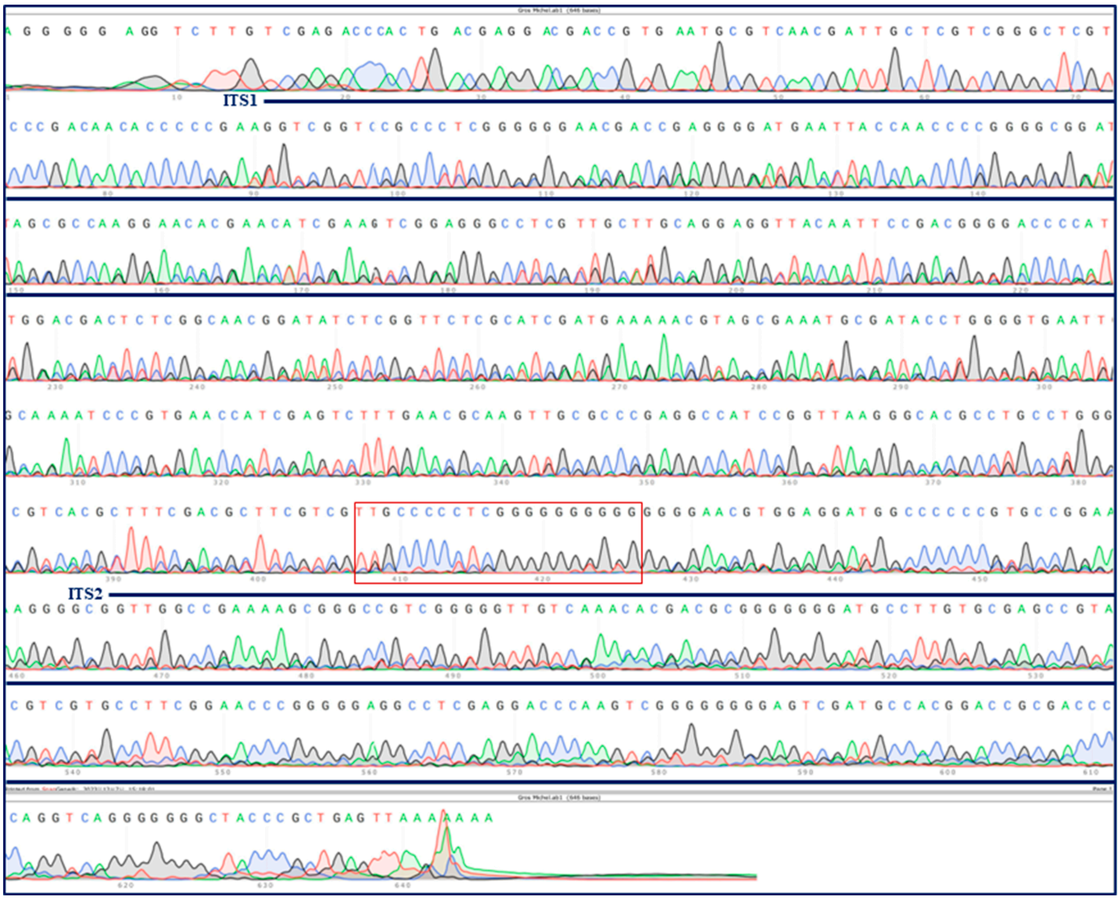

Supplemental Figure S6. ITS sequencing peak map of the Gros Michel banana Gros Michel variety. ITS of 'Gros Michel' were amplified by PCR and sequenced through Sanger sequencing. The positions of ITS1, ITS2 and the "420-bp region" are indicated.

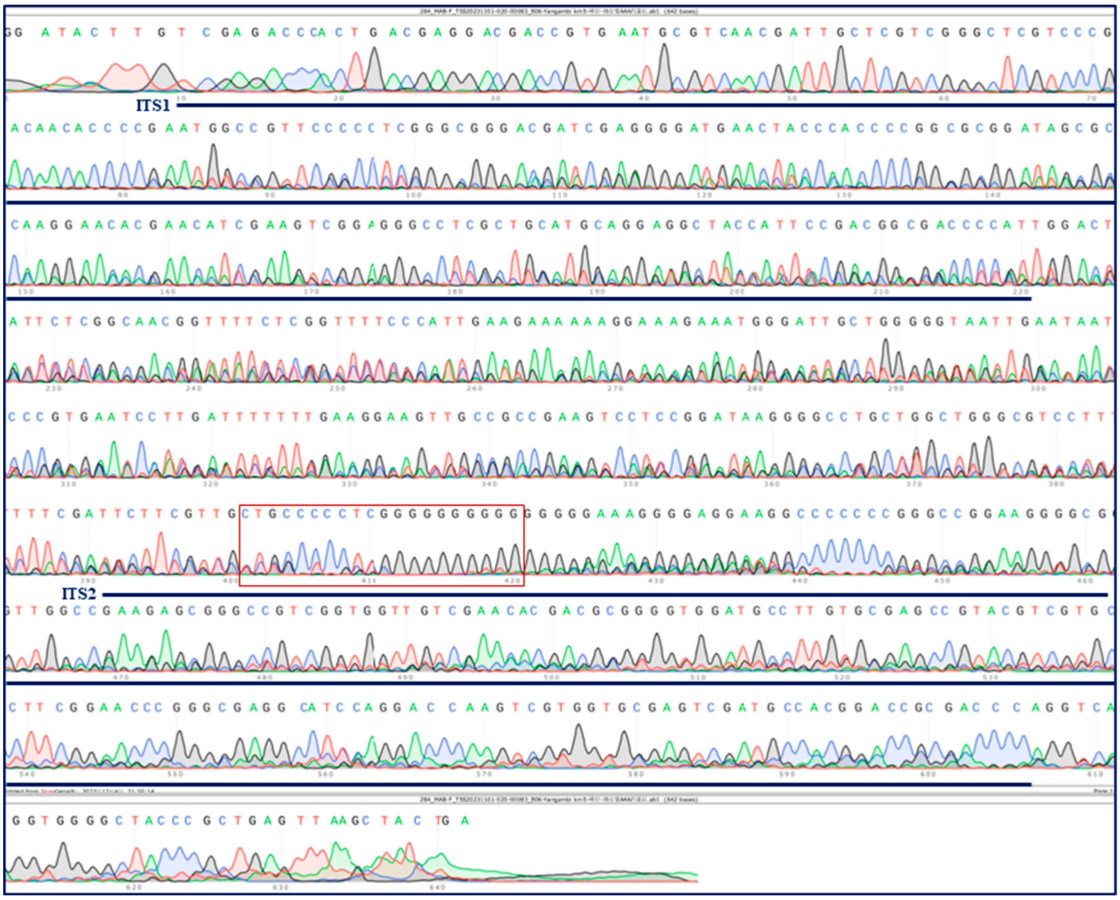

Supplemental Figure S7. ITS sequencing peak map of the Ibota Bota banana Yangambi KM5 variety. ITS of ‘Yangambi KM5’ were amplified by PCR and sequenced through Sanger sequencing. The positions of ITS1, ITS2 and the “420-bp region” are indicated.

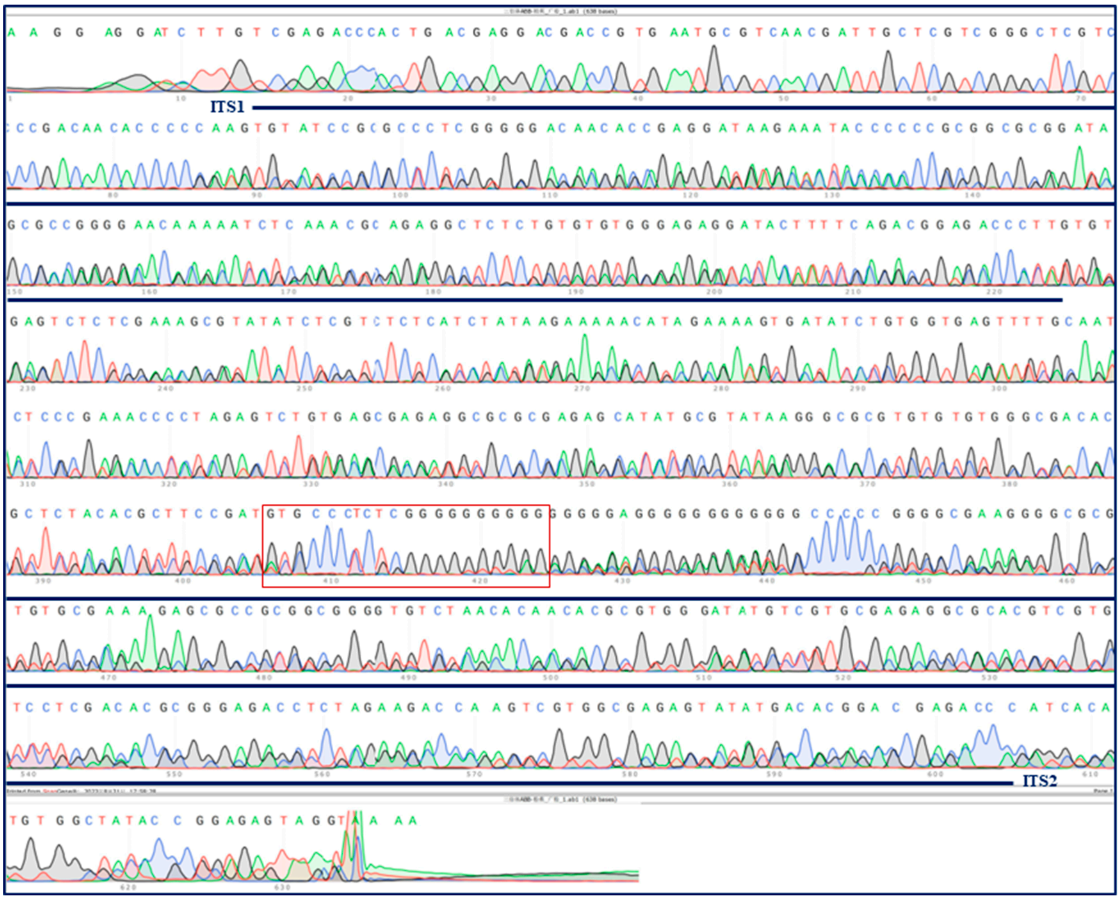

Supplemental Figure S8. ITS sequencing peak map of the Pisang Awak banana Guang Fen No.1 variety. ITS of ‘Guang Fen No.1’ were amplified by PCR and sequenced through Sanger sequencing. The positions of ITS1, ITS2 and the “420-bp region” are indicated.

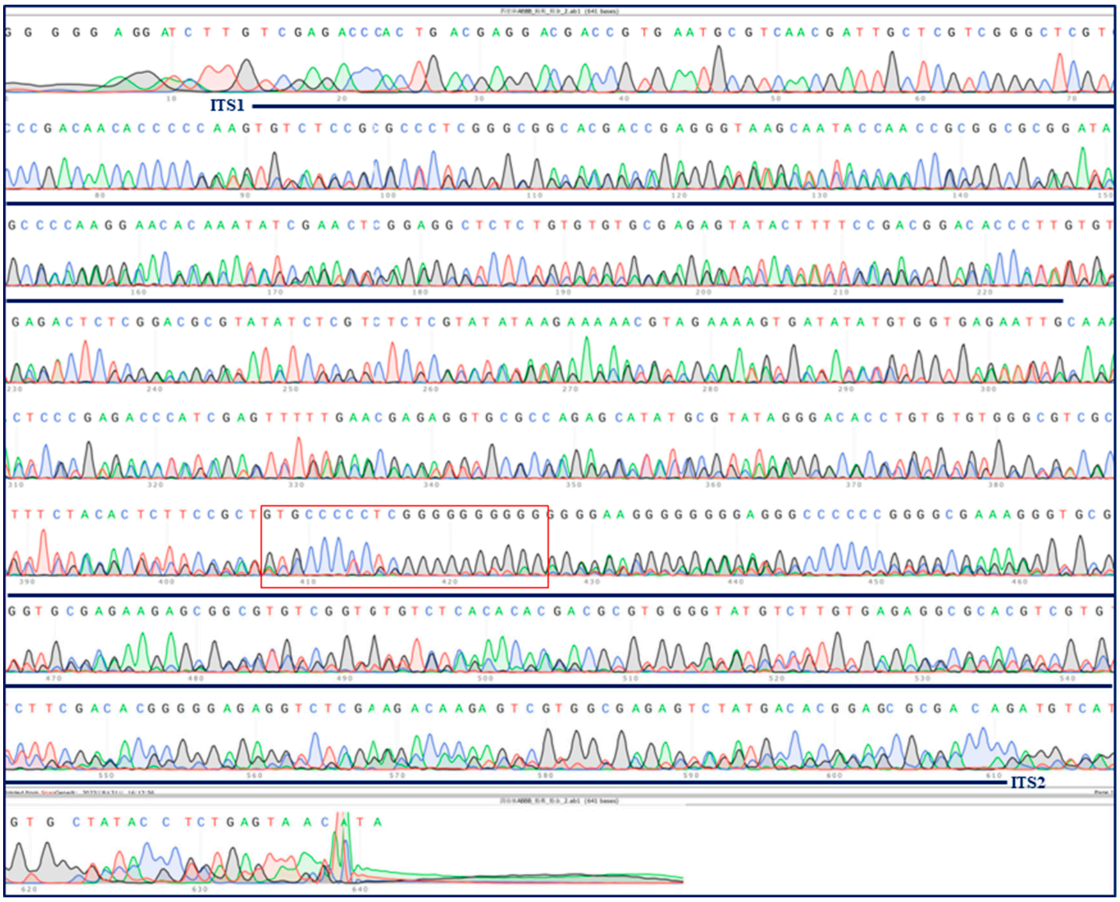

Supplemental Figure S9. ITS sequencing peak map of the hybrid banana Fen Za No.1 variety. ITS of 'Fen Za No.1' were amplified by PCR and sequenced through Sanger sequencing. The positions of ITS1, ITS2 and the "420-bp region" are indicated.

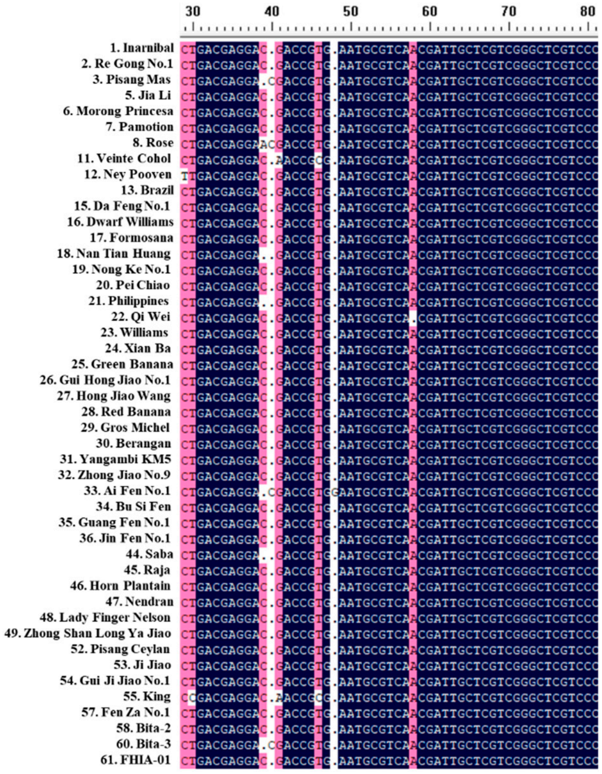

Supplemental Figure S10. Sequence alignment of the partial ITS regions across 46 accessions of banana. The initial several dozen bases of the ITS region from the 46 accessions represent relatively homozygous sequences, which were aligned accordingly.

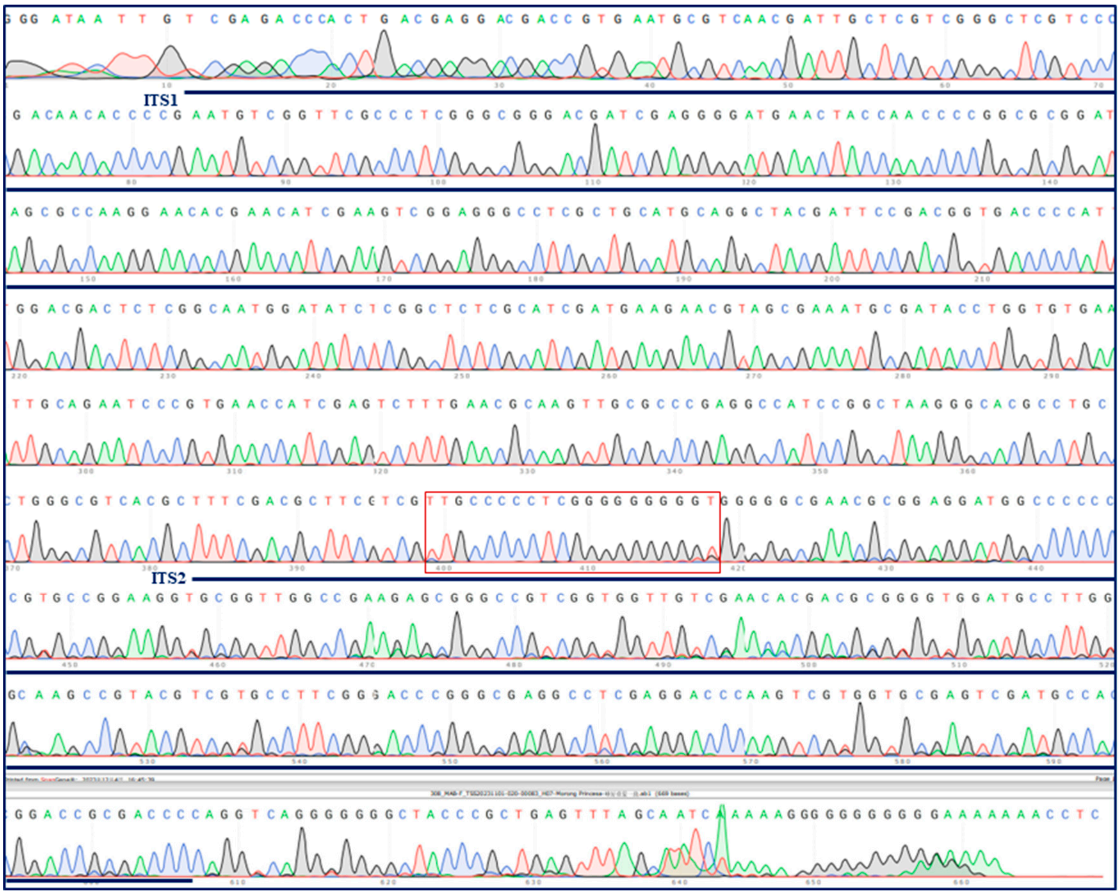

Supplemental Figure S11. ITS sequencing peak map of the AA banana Morong Princesa variety. ITS of ‘Morong Princesa’ were amplified by PCR and sequenced through Sanger sequencing. The positions of ITS1, ITS2 and the “420-bp region” are indicated.

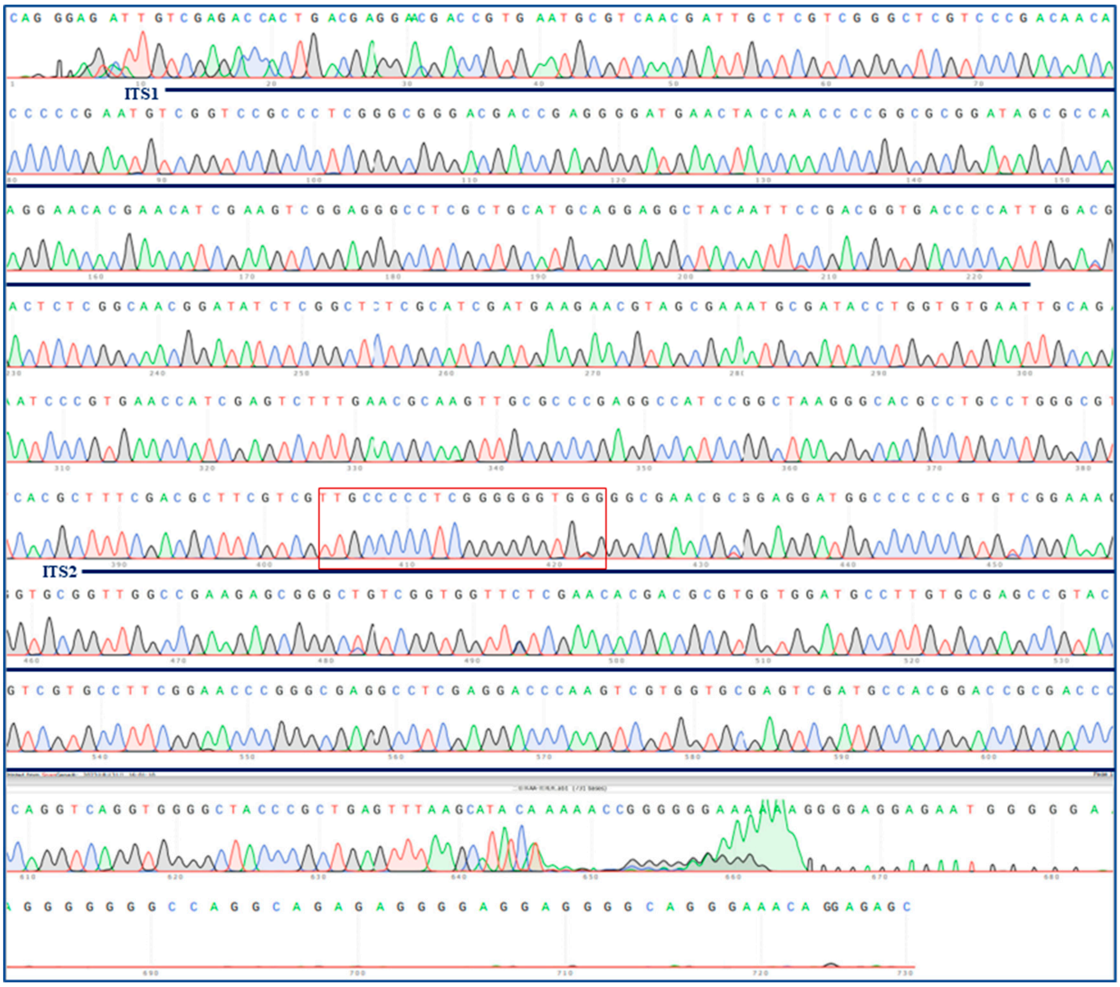

Supplemental Figure S12. ITS sequencing peak map of the AA banana Rose variety. ITS of 'Rose' were amplified by PCR and sequenced through Sanger sequencing. The positions of ITS1, ITS2 and the "420-bp region" are indicated.

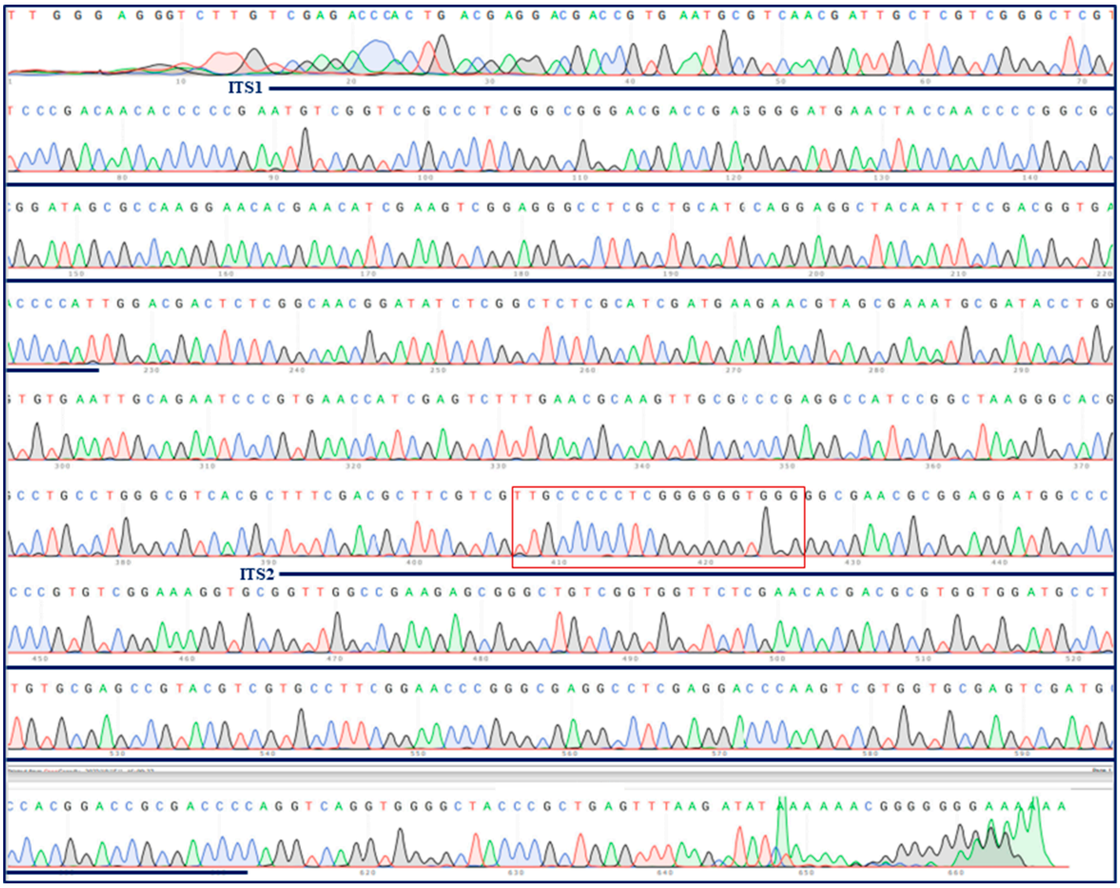

Supplemental Figure S13. ITS sequencing peak map of the hybrid banana Zhong Jiao No.9 variety. ITS of ‘Zhong Jiao No.9’ were amplified by PCR and sequenced through Sanger sequencing. The positions of ITS1, ITS2 and the “420-bp region” are indicated.

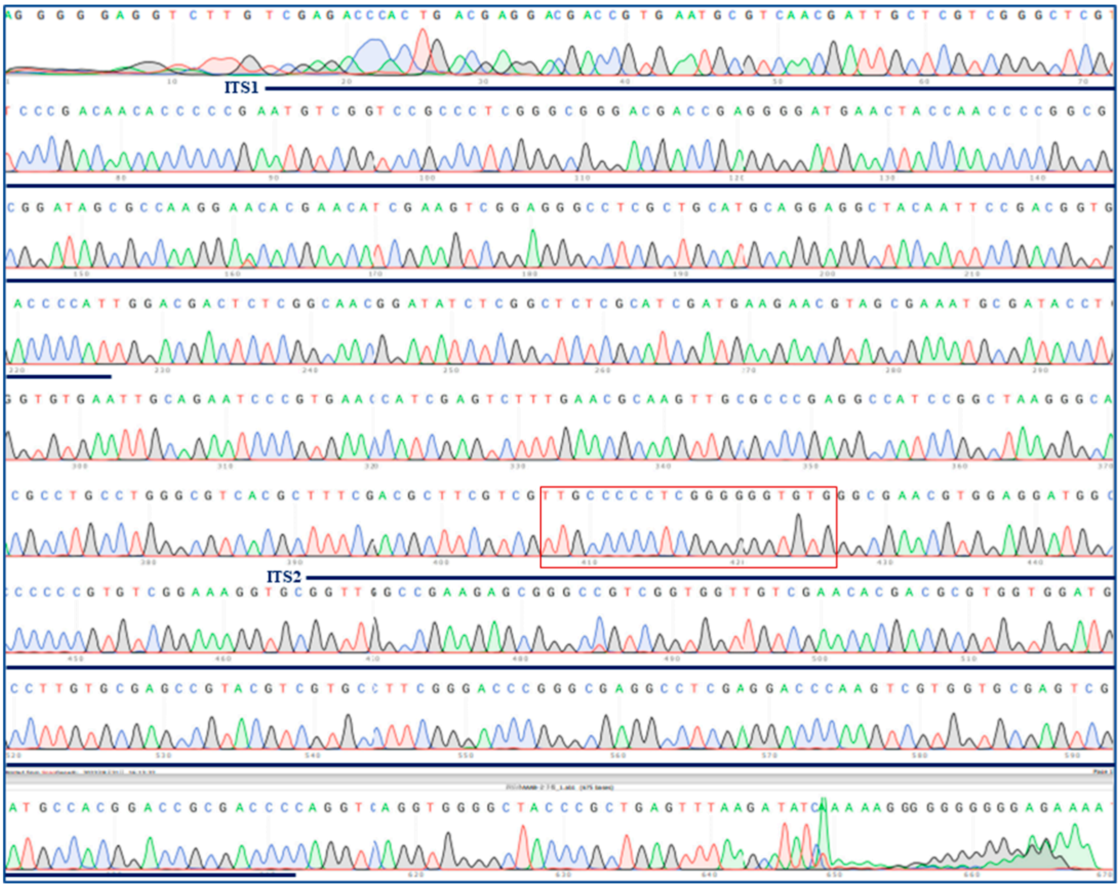

Supplemental Figure S14. ITS sequencing peak map of the hybrid banana FHIA-01 variety. ITS of ‘FHIA-01’ were amplified by PCR and sequenced through Sanger sequencing. The positions of ITS1, ITS2 and the “420-bp region” are indicated.

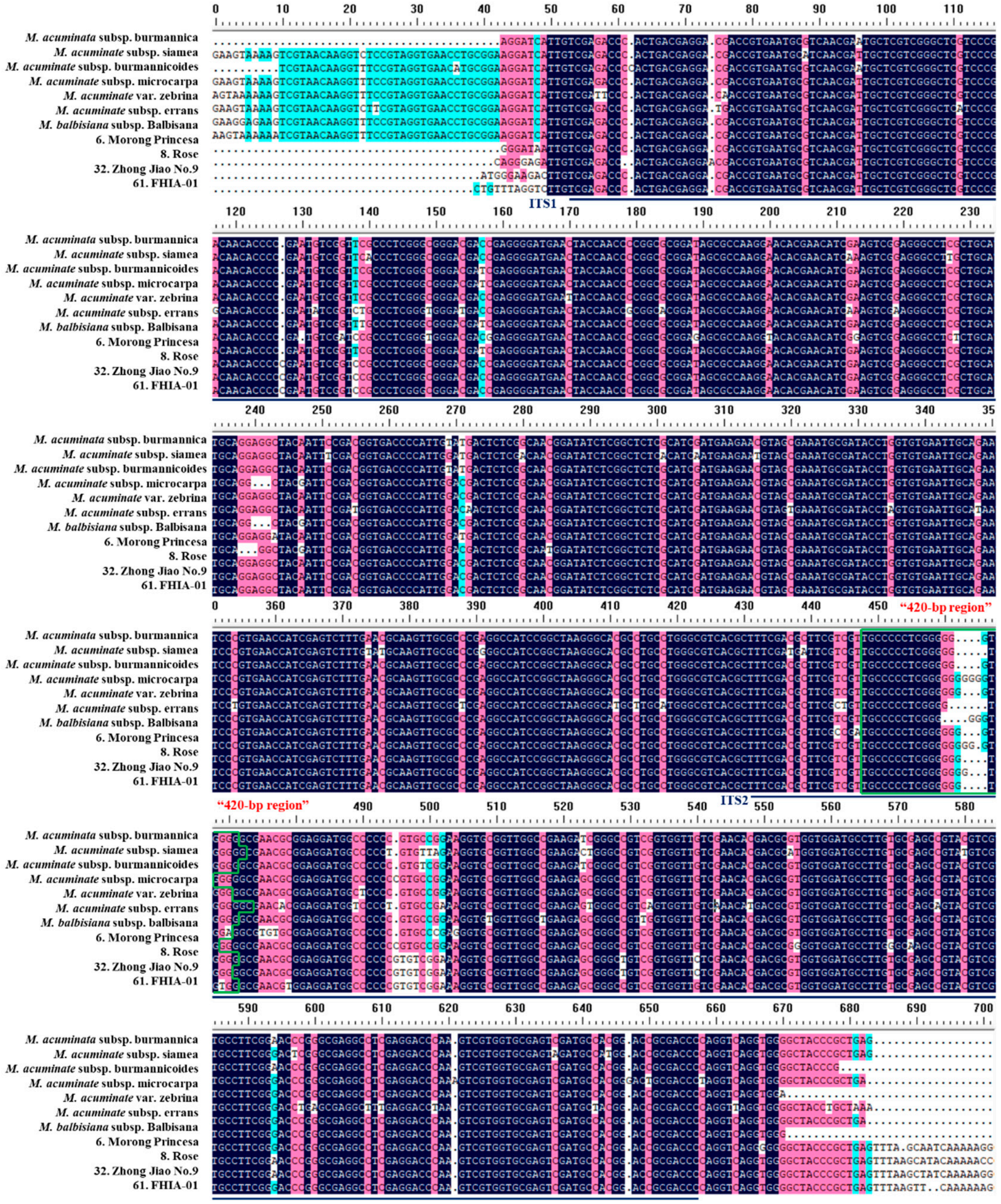

Supplemental Figure S15. Sequence alignment of the ITS region from 7 wild banana species and 4 cultivated banana varieties. The ITS sequences of the wild bananas were obtained from NCBI. The positions of ITS1, ITS2 and the "420-bp region" are indicated.
